# Synthesis and Biological Activity of Arylspiroborate Salts Derived from Caffeic Acid Phenethyl Ester

**DOI:** 10.1155/2015/418362

**Published:** 2015-03-05

**Authors:** Martin J. G. Hébert, Andrew J. Flewelling, Trevor N. Clark, Natalie A. Levesque, Jacques Jean-François, Marc E. Surette, Christopher A. Gray, Christopher M. Vogels, Mohamed Touaibia, Stephen A. Westcott

**Affiliations:** ^1^Department of Chemistry and Biochemistry, Université de Moncton, Moncton, NB, Canada E1A 3E9; ^2^Department of Biology, University of New Brunswick, Saint John, NB, Canada E2L 4L5; ^3^Department of Chemistry, University of New Brunswick, Fredericton, NB, Canada E3B 5A3; ^4^Department of Chemistry and Biochemistry, Mount Allison University, Sackville, NB, Canada E4L 1G8

## Abstract

Two novel boron compounds containing caffeic acid phenethyl ester (CAPE) derivatives have been prepared and characterized fully. These new compounds and CAPE have been investigated for potential antioxidant and antimicrobial properties and their ability to inhibit 5-lipoxygenase and whether chelation to boron improves their biological activity. Sodium salt **4** was generally more active than ammonium salt **5** in the biological assays and surpassed the radical scavenging ability of CAPE. Compounds **4** and **5** were more active than CAPE and Zileuton in human polymorphonuclear leukocytes. These results clearly show the effectiveness of the synthesized salts as transporter of CAPE.

## 1. Introduction

**Figure 1:**
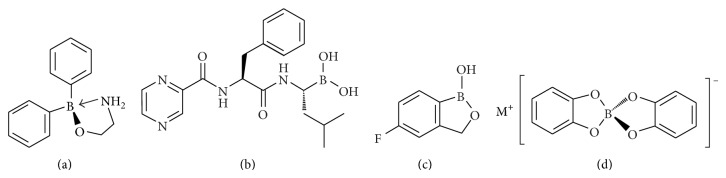
Structures of 2-aminoethoxydiphenyl borate (a), Velcade (b), AN2690 (c), and arylspiroborate salts (d).

Compounds containing boron groups have attracted considerable interest recently owing to their extensive use as synthons in organic synthesis, particularly in Suzuki cross coupling reactions [1]. Organoboronic acids [RB(OH)_2_] and boronate esters [RB(OR′)_2_] are exceptionally attractive for these carbon-carbon bond forming reactions as they are generally readily prepared, are air- and water-stable, and have relatively low toxicities. Interest in boron compounds also arises, however, from the discovery that these simple compounds can display a wide range of biological properties [2]. For instance, 2-aminoethoxydiphenyl borate (Figure 1(a)) is a common activator of the transient receptor potential cation channel subfamily V members (TRPV1, TRPV2, and TRPV3); and while chemical activators for TRPV1 are common, only a few examples are known to activate TRPV2 and TRPV3 [3]. The first example of a boron compound approved for clinical trials is Velcade (Figure 1(b)) used for the treatment of multiple myeloma [4]. Another example of a simple bioactive boron-containing compound is the benzoxaborole compound AN2690 (Figure 1(c)), which displays potent antifungal properties [5]. Likewise, more relevant to this work is the observation that arylspiroborates also display antifungal properties and are used for the treatment of wood against parasites such as termites [6].

**Figure 2:**
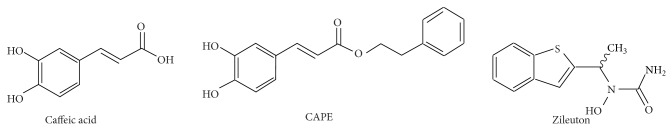
The molecular structures of caffeic acid, CAPE, and Zileuton.

We have begun to investigate the synthesis and bioactivity of arylspiroborate salts derived from substituted catechols. Indeed, we have found that boron salts derived from 4-*tert*-butylcatechol and 3,5-di-*tert*-butylcatechol had moderate antimicrobial activities [7]. As part of our study, we generated the corresponding arylspiroborate salts from caffeic acid phenethyl ester (CAPE). Caffeic acid (Figure 2) is the major component in hydroxycinnamic and phenolic acids and is naturally found in plants in a variety of forms, including the corresponding esters. The chemistry and medicinal potential of CAPE derivatives have been well-documented [8], displaying potent antioxidant, anticancer, and antifungal properties [9, 10]. CAPE was also reported as an interesting 5-lipoxygenase (5-LO) inhibitor compared to Zileuton [11, 12]. The latter is an antiasthmatic drug [13] and the only 5-LO inhibitor available for use in clinical or commercial settings although severe side effects have limited its use [14]. 5-LO is the main enzyme regulating the synthesis of leukotrienes (LTs) from arachidonic acid (AA). LTs are key lipid mediators of various inflammatory processes such as atherosclerosis [15], asthma [16], and rheumatoid arthritis [17]. In this study we report the synthesis of boron derivatives of CAPE along with initial bioactivity testing, where it was hoped that the salt form would aid in delivery and enhance activities.

## 2. Results and Discussion

### 2.1. Chemistry

**Scheme 1:**
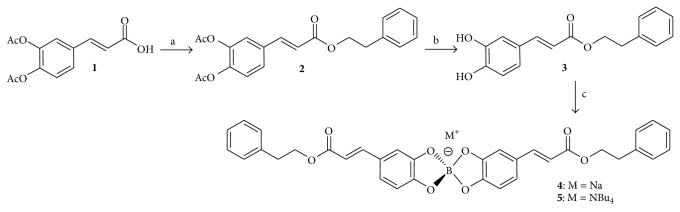
Synthesis of CAPE (**3**) and arylspiroborate salts** 4** and** 5**.* Reagents and Conditions*. (a) (i) SOCl_2_, reflux, 1 h; (ii) HOCH_2_CH_2_Ph, toluene, pyridine, rt, 12 h. Yield: 78%. (b) Guanidine hydrochloride, CH_3_OH–CH_2_Cl_2_ (1 : 1), rt, 4 h. Yield: 79%. (c) MBH_4_, CH_3_OH, 6 h (1 h for** 5**). Yields: 85% for** 4**, 76% for** 5**.

As described previously for the CAPE related propionic, acetic, and benzoic acid analogues [8], ester** 2** was synthesized from 2-phenylethanol with acetylated caffeic acid** 1**. The conversion of** 1** into the corresponding carboxylic chloride was achieved by the Vilsmeier-Haack adduct [18] derived from thionyl chloride [19] and* N*,*N*-dimethylformamide (DMF) as catalyst. Deacetylation of compound** 2** to afford CAPE (**3**) was performed with guanidine hydrochloride and triethylamine in MeOH/CH_2_Cl_2_ [20]. Addition of two equivalents of** 3** to solutions of commercially available borohydride salts in methanol afforded the corresponding arylspiroborates in good yields along with the formation of dihydrogen (Scheme 1). The boron salts were characterized by a number of physical methods, including multinuclear NMR spectroscopy. The ^11^B NMR spectra for these compounds show a sharp singlet at approximately 14 ppm, signifying that the boron atom remains in a four-coordinate environment. Little change is observed by either ^1^H or ^13^C NMR spectroscopy upon chelation of the organic CAPE group to the boron atom. Although this methodology could be used to generate a number of salts, we decided to focus on sodium salt** 4** and the tetrabutylammonium analogue** 5** but, unfortunately, the lipophilic butyl groups had only a negligible effect on improving the solubility of this salt in organic solvents. With these new arylspiroborates elementally pure and owing to the wealth of bioactivities associated with simple boron compounds, we then decided to examine these new CAPE derivatives for their potential to act as antioxidant and antimicrobial agents.

### 2.2. Bioactivities

#### 2.2.1. Antioxidant Activity

Radical scavenging activities of CAPE (**3**) and compounds** 4** and** 5** were assayed using 2,2-diphenyl-1-picrylhydrazyl (DPPH) as a stable radical [21] and are expressed as IC_50_ concentrations in Table 1. As was expected of catechol-containing molecules all compounds possessed radical scavenging ability with most having IC_50_ values in the range of 9.5–15 *μ*M. As shown in Table 1, sodium salt** 4** is more active than ammonium salt** 5** and surpasses the radical scavenging ability of CAPE (**3**) and of Zileuton which is almost nonactive.

#### 2.2.2. Antimicrobial Activity

As mentioned previously, arylspiroborates are known to display significant antimicrobial properties. With this in mind, we examined the inhibitory activity of CAPE (**3**) and compounds** 4** and** 5** against* Candida albicans*,* Pseudomonas aeruginosa*,* Staphylococcus aureus,* and methicillin-resistant* S. aureus* (MRSA) using boric acid as a control to see what role, if any, the boron atom has on bioactivity. Although the new salts did not display any significant activity against the Gram-negative bacterium* P. aeruginosa* in our initial screening (growth inhibition ranged from 5 to 25% at 100 *μ*g/mL), these compounds did show some weak antifungal activity against* C. albicans* and modest activity against the Gram-positive bacterium* S. aureus* and MRSA (see Table 2). Studies with the ammonium salt** 5** were complicated by the poor solubility of this salt in physiological media and the data obtained for this complex should therefore be interpreted with caution. It is, however, interesting to note that although** 5** was not significantly active against* S. aureus* (causing only 31% inhibition when screened at 100 *μ*g/mL), the activity of** 4** was comparable to that of CAPE (**3**). In contrast, CAPE (**3**) was considerably more active against the methicillin-resistant* S. aureus* than both** 4** and** 5**, which exhibited similar activity against MRSA.

#### 2.2.3. 5-LO Inhibition

**Figure 3:**
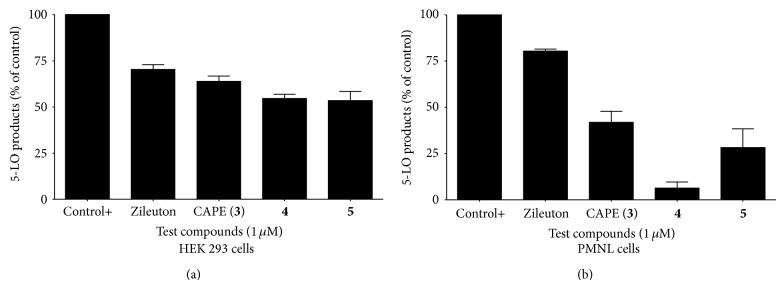
Inhibition of 5-LO products biosynthesis by Zileuton, CAPE (**3**), sodium salt** 4**, and ammonium salt** 5** in HEK293 and PMNL cells. Values are means ± SEM.

The inhibitory capacities of synthesized sodium and ammonium salts were at first analyzed in double stable transfectants of intact HEK 293 cells expressing both 5-LO and 5-LO activating protein (FLAP), thereby possessing all the necessary cellular machinery required for LTs biosynthesis [22]. Zileuton and CAPE were used as reference. As shown in Figure 3, compounds** 4** and** 5** have similar 5-LO products biosynthesis inhibitory activity in intact HEK293 cells as Zileuton and CAPE (**3**) at concentrations of 1 *μ*M. This result clearly shows the effectiveness of the synthesized boron salts as transporter of CAPE, where a total or even partial hydrolysis occurs for the release of CAPE (**3**). Indeed, the formation of CAPE (**3**) under these biological conditions was confirmed using HPLC and UV-vis spectroscopy (see Section 3.4). Likewise, a peak in the ^11^B NMR spectrum for salt** 4** was observed in DMSO-d_6_ after one hour along with significant amounts of a peak at 19 ppm for degradation product boric acid, B(OH)_3_. The stability of these salts was examined using representative salt** 5**, but compound** 4** showed similar reactivity (see Section 3.4).

In order to further probe inhibitory capacity of our sodium** 4** and ammonium salts** 5**, tests were undertaken to investigate inhibition of 5-LO in stimulated human polymorphonuclear leukocytes (PMNL) cells in the presence of 1 *μ*M of the test compounds. 5-LO is highly expressed in PMNL which are important producers of LTB4 [22, 23]. As seen in Figure 3, sodium salt** 4** is the more active of the compounds tested and more potent than the only known inhibitor for commercial and clinical uses, Zileuton. The more efficacious inhibitory effect of** 4** may be due to its more effective uptake into PMNL.

Concentration-response studies in thapsigargin-stimulated human PMNL were investigated to allow a better insight into the potency of the compounds. The results are presented in Table 3. Compounds** 4** and** 5** inhibited 5-LO products synthesis in a concentration-dependent manner in human PMNL and showed prominent inhibitory activities with IC_50_ values ranging from 0.6 to 0.8 *μ*M. Sodium salt** 4** was clearly the more potent being more active than both Zileuton and CAPE (**3**) (5 and 1.8 times, resp.). Ammonium salt** 5** was also more active than Zileuton and CAPE (**3**) (3.8 and 1.3 times, resp.).

## 3. Experimental

### 3.1. General Procedures

Reagents and solvents used were obtained from Aldrich Chemicals. NMR spectra for** 1**–**3** were recorded on a Bruker Avance III 400 MHz spectrometer and mass spectrometric measurements were performed on a MicrOTOF instrument from Bruker Doltonics in positive electrospray. NMR spectra for** 4** and** 5** were recorded on a JEOL JNM-GSX400 FT NMR (^1^H: 400 MHz; ^11^B: 128 MHz; ^13^C: 100 MHz) spectrometer. The ppm scale was calibrated using the residual solvent signal peaks (^1^H and ^13^C) and external BF_3_·OEt_2_(^11^B). Multiplicities are reported as singlet (s), doublet (d), triplet (t), sextet (sext), multiplet (m), broad (br), and overlapping (ov). FT-IR spectra were obtained with a Thermo Fisher Scientific Nicolet iS5 FT-IR spectrometer in ATR mode and are reported in cm^−1^. Melting points were determined using a Stuart SMP30 melting point apparatus and are uncorrected. Elemental analyses for carbon, hydrogen, and nitrogen were carried out at Laboratoire d'Analyse Élémentaire de l'Université de Montréal (Montréal, QC). Reactions were performed under an atmosphere of dinitrogen. TLC was performed on Kieselgel 60 F254 plates from Merck. Detection was carried out under UV light (254 nm) or by molybdate solution (235 mL of distilled water, 12 g of ammonium molybdate, 0.5 g of ceric ammonium molybdate, and 15 mL of concentrated sulfuric acid) followed by heating. Separations were carried out on silica gel (7749 Merck) using circular chromatography (Chromatotron, model 7924, Harrison Research). Melting points of the arylspiroborate salts were obtained using a MEL-TEMP (model 1001D) melting point apparatus. Either protonated ions (M + H)^+^ or sodium adducts (M + Na)^+^ were used for empirical formula confirmation.

### 3.2. Chemistry

#### 3.2.1. Synthesis of Acetylated CAPE (**2**)

A mixture of diacetylcaffeic acid [24] (250 mg, 0.94 mmol), 10 mL of thionyl chloride, and two drops of DMF was heated at reflux for 4 h. The excess thionyl chloride was removed on a Rotovap, and the residue is dissolved in 4 mL of dry dichloromethane. To this solution is slowly added 1 mL of pyridine and 2-phenylethanol (127.2 mg, 125 *μ*L, 1.04 mmol, 1.1 eq). The resulting mixture was stirred overnight at room temperature. After removal of solvents, the residue was dissolved in ethyl acetate (25 mL); the organic extract was washed with water (2 × 20 mL) and brine (2 × 20 mL) and then dried over MgSO_4_. Purification by silica gel circular chromatography (1–20% EtOAc–hexane) gave acetylated CAPE** 2** (272 mg, 78%) as a white solid (mp 80–82°C). Rf = 0.45 (30% EtOAc-hex); IR (*ν*, cm^−1^): 3037, 2930, 1774, 1713, 1642, 1504, 1204. Spectroscopic NMR data: ^1^H (CDCl_3_); *δ* (ppm) d 7.51 (d, 1H,* J* = 15.9 Hz, CHC_ar_), 7.42–7.23 (m, 8H, H_ar_), 6.38 (d, 1H,* J* = 15.9 Hz, CHCO), 4.44 (t, 2H,* J* = 7 Hz, CH_2_O), 3.04 (t, 2H,* J* = 7 Hz, CH_2_O), 2.33 (s, 6H, 2 OAc). ^13^C (CDCl_3_); *δ* (ppm) 168.08, 167.98, 166.49, 143.50, 142.90, 142.44, 137.81, 133.30, 128.93, 128.55, 126.61, 126.43, 123.93, 122.71, 119.25, 65.16, 35.18, 20.66, 20.62; HRMS* m/z* calcd for C_21_H_20_O_6_^+^(Na^+^): 391.1152; found: 391.1151.

#### 3.2.2. Synthesis of CAPE (**3**)

The acetylated CAPE (**2**) (200 mg, 0.54 mmol) was dissolved in 2 mL of anhydrous CH_2_Cl_2_ under N_2_, to which 4 mL of MeOH was added. Guanidinium hydrochloride (168.5 mg, 1.76 mmol, and 3.25 eq) and triethylamine (631.7 mg, 738 *μ*L, 5.26 mmol, and 9.75 eq) were added to the stirred solution. After consumption of the diacetylated precursor (about 2 h), the reaction mixture was concentrated and partitioned between 60 mL of AcOEt and 30 mL of water. The organic phase was washed sequentially with water (50 mL), saturated ammonium chloride (2 × 20 mL), and brine (2 × 20 mL), treated with charcoal, dried over MgSO_4_, and concentrated to give pure CAPE (**3**) (150 mg, 79%) of a white solid (mp 126–128°C); Rf = 0.37 (50% EtOAc-hexane); IR (*ν*, cm^−1^): 3330, 3058, 2986, 1678, 1596, 1265. Spectroscopic NMR data: ^1^H (CDCl_3_); *δ* (ppm) 9.60 (s, 1H, OH), 9.13 (s, 1H, OH), d 7.45 (d, 1H,* J* = 15.8 Hz, CHC_Ar_), 7.33–7.21 (m, 5H, H_Ar_), 7.04 (s, 1H, H_Ar_), 6.99 (d, 1H,* J* = 8.2 Hz, H_Ar_), 6.76 (d, 1H,* J* = 8.1 Hz, H_Ar_), 6.23 (d, 1H,* J* = 15.8 Hz, CHCO), 4.32 (t, 2H,* J* = 6.8 Hz, CH_2_O), 2.95 (t, 2H,* J* = 6.8 Hz, CH_2_O); ^13^C (CDCl_3_); *δ* (ppm) 166.93, 148.90, 146.03, 145.68, 138.57, 129.35, 128.83, 126.83, 125.91, 121.85, 116.20, 115.31, 114.29, 64.77, 34.96; HRMS* m/z* calcd for C_17_H_16_O_4_^+^(Na^+^): 307.0941; found: 307.0934.

#### 3.2.3. Synthesis of Sodium 5,5′-Bis((E)-3-oxo-3-phenethoxyprop-1-en-yl)-2,2′-spirobi[benzo[d][1,3,2]dioxaborol]-2-uide (**4**)

Sodium borohydride (14 mg, 0.37 mmol) was added in small portions as a solid to a stirred THF (5 mL) solution of CAPE (**3**) (210 mg, 0.74 mmol). The reaction mixture was heated at reflux for 6 h at which point hexane (5 mL) was added to the clear yellow solution to afford a white precipitate. The precipitate was collected by suction filtration and washed with hexane (3 × 1 mL) to afford** 4** as a white solid. Yield: 188 mg (85%); mp: 300–303°C; IR (*ν*, cm^−1^): 3056, 3027, 1697, 1682, 1628, 1607, 1593, 1489, 1450, 1316, 1252, 1238, 1183, 1146, 1099, 1052, 971, 921, 850, 808, 749, 696, 607. Spectroscopic NMR data: ^1^H (DMSO-d_6_); *δ* (ppm) 7.45 (d,* J* = 16.0 Hz, 2H, CH=CH), 7.30–7.25 (ov m, 8H, Ar), 7.19 (m, 2H, Ar), 6.91 (d,* J* = 1.4 Hz, 2H, Ar), 6.84 (dd,* J* = 7.8, 1.4 Hz, 2H, Ar), 6.51 (d,* J* = 7.8 Hz, 2H, Ar), 6.21 (d,* J* = 16.0 Hz, 2H, CH=CH), 4.27 (t,* J* = 6.8 Hz, 4H, CH_2_CH_2_), 2.91 (t,* J* = 6.8 Hz, 4H, CH_2_CH_2_); ^11^B (THF): *δ* (ppm) 13.5 (sharp); ^13^C (DMSO-d_6_): *δ* (ppm) 167.4 (O–C=O), 155.3, 152.5, 147.2, 138.7, 129.4, 128.9, 126.9, 124.9, 123.2, 112.6, 108.3, 106.2, 64.7 (OCH_2_CH_2_), 35.1 (OCH_2_CH_2_). Anal. Calcd. for C_34_H_28_BNaO_8_ (598.38): C, 68.24; H, 4.72. Found: C, 67.94; H, 4.57.

#### 3.2.4. Synthesis of Tetrabutylammonium 5,5′-Bis((E)-3-oxo-3-phenethoxyprop-1-en-yl)-2,2′-spirobi[benzo[d][1,3,2]dioxaborol]-2-uide (**5**)

Tetrabutylammonium borohydride (91 mg, 0.35 mmol) was added in small portions as a solid to a stirred Et_2_O (5 mL) solution of CAPE (**3**) (200 mg, 0.70 mmol). The reaction was allowed to proceed for 1 h at which point a white precipitate was collected by suction filtration. The precipitate was washed with Et_2_O (3 × 1 mL) to afford** 5** as a white solid. Yield: 218 mg (76%); mp: 42–45°C. IR (*ν*, cm^−1^): 2961, 2874, 1696, 1625, 1588, 1487, 1453, 1382, 1311, 1237, 1167, 1148, 1117, 1089, 1043, 980, 897, 846, 806, 749, 698, 609. Spectroscopic NMR data: ^1^H (DMSO-d_6_): *δ* (ppm) 7.45 (d,* J* = 16.0 Hz, 2H, CH=CH), 7.30–7.25 (ov m, 8H, Ar), 7.20–7.17 (ov m, 2H, Ar), 6.91 (d,* J* = 1.4 Hz, 2H, Ar), 6.84 (dd,* J* = 7.8, 1.4 Hz, 2H, Ar), 6.51 (d,* J* = 7.8 Hz, 2H, Ar), 6.21 (d,* J* = 16.0 Hz, 2H, CH=CH), 4.27 (t,* J* = 6.9 Hz, 4H, OCH_2_CH_2_), 3.09 (br m, 8H, NCH_2_), 2.91 (t,* J* = 6.8 Hz, 4H, OCH_2_CH_2_), 1.50 (br m, 8H, –CH_2_–), 1.25 (sext,* J* = 6.8 Hz, 8H, CH_2_CH_3_), 0.87 (t,* J* = 6.8 Hz, 12H, CH_3_). ^11^B (THF): *δ* (ppm) 13.5 (sharp). ^13^C (DMSO-d_6_): *δ* (ppm) 167.4 (O–C=O), 155.3, 152.4, 147.2, 138.7, 129.4, 128.9, 126.9, 124.9, 123.2, 112.6, 108.3, 106.2, 64.7 (OCH_2_CH_2_), 58.0 (NCH_2_), 35.1 (OCH_2_CH_2_), 23.7 (NCH_2_CH_2_), 19.6 (CH_2_CH_3_), 13.7 (CH_3_). Anal. Calcd. for C_50_H_64_NBO_8_ (817.86): C, 73.43; H, 7.89; N, 1.71. Found: C, 73.98; H, 7.97; N, 1.67.

### 3.3. Bioassays

#### 3.3.1. In Vitro Antioxidant Activity

Testing compounds to scavenge radicals was evaluated by the DPPH assay [18]. To avoid discrepancies in IC_50_ calculation, meticulous care was applied in the preparation of the control solution (DPPH reagent + ethanol as a diluent without test compounds). Only controls showing an optical density (OD) of 0.350–0.360 were used. 1 mL of the DPPH reagent diluted in methanol (60 mM) was added to 1 mL of the test compound at various concentrations. After vigorous vortexing, incubation was performed in the dark for 30 min. OD measurements were done at 520 nm. Radical scavenging was termed as % inhibition of DPPH absorbance: % Inhibition = [(*A*_control_ − *A*_test_)/*A*_control_] × 100, where *A*_control_ is the OD of the control (DPPH solution in absence of test compound) and *A*_test_ is the absorbance of the test sample (DPPH solution in presence of test compound). The data presented are means of two experiments, each performed in triplicate. GraphPad Prism 5 software (GraphPad Software, San Diego, CA, USA) was used to calculate IC_50_ values by fitting experimental values to a sigmoidal concentration-response curve-fitting model with a variable slope.

#### 3.3.2. Antibiotic Susceptibility Assays

Antibacterial activity against* Staphylococcus aureus* (ATCC 29213), MRSA (ATCC 33591), and* Pseudomonas aeruginosa* (ATCC 10145) and antifungal activity against* Candida albicans* (ATCC 14053) were evaluated using a microbroth dilution antibiotic susceptibility assay modified from Webb et al. [7, 19]. Stock solutions of the test compounds (1.25 mg/mL) were prepared with sterile-filtered DMSO, stored at 4°C, and used within one week of preparation. Immediately prior to use, stock solutions (40 *μ*L) were diluted with the appropriate nutrient broth (960 *μ*L) and the resulting test solutions (100 *μ*L) were transferred to nonperipheral wells of a 96-well microtitre plate in triplicate. Wells were then inoculated with suspensions of the appropriate microbial strain (100 *μ*L) of cell density 5 × 10^5^ colony forming units/mL and to reduce evaporation from the plates, sterile water (200 *μ*L) was added to perimeter wells. Positive controls consisted of a triplicate twofold serial dilution of antibiotic (1.25–0.313 *μ*g/mL, gentamicin for* S. aureus*; 5–1.25 *μ*g/mL, gentamicin for* P. aeruginosa*; 10–2.5 *μ*g/mL, nystatin for* C. albicans*; 0.2–0.05 *μ*g/mL, ciprofloxacin for MRSA; 100 *μ*L per well) as 4% DMSO solutions in the appropriate nutrient broth inoculated with suspensions of the appropriate pathogen (100 *μ*L). In addition to test and positive control wells, each plate contained three untreated controls [4% DMSO in the appropriate nutrient broth (100 *μ*L) inoculated with suspensions of the appropriate pathogen (100 *μ*L)] and three uninoculated blanks (200 *μ*L of 2% DMSO in the appropriate nutrient broth). The optical densities of the wells were measured before and after a 24-hour incubation period (37°C), and the change in optical density (ΔOD) was calculated by subtracting the initial optical density from the final optical density of corresponding wells. ΔOD values were corrected for background absorbance of the media by subtracting the mean ΔOD readings of the blanks from the mean ΔOD readings of the control and test compound wells. The percentage inhibition of bacterial or fungal growth was then defined as follows: [1 − (mean test or positive control ΔOD/mean negative control ΔOD)] × 100.

MIC and IC_50_ values were determined for test compounds that exhibited >50% growth inhibition at a concentration of 100 *μ*g/mL in the initial screening assays, which were performed as described above on twofold serial dilutions of active compounds in triplicate. All compounds tested against* C. albicans* were tested at 10 concentrations obtained from two dilution series (200.0–12.5 *μ*g/mL and 150.0–9.38 *μ*g/mL). All compounds tested against* S. aureus* and MRSA were tested at 15 concentrations obtained from two dilution series (200.0–0.39 *μ*g/mL and 150.0–9.38 *μ*g/mL). The MIC of a compound was considered to be the lowest assay concentration at which it inhibited mycobacterial growth by more than a mean value of 90% [20]. Absolute IC_50_s were estimated by fitting a four-parameter logistic curve to the microbial growth data (in relative fluorescence or OD units) [21] using Masterplex 2010 Readerfit (Hitachi). In cases when the data were insufficient to obtain reliable estimates by four-parameter logistic regression, absolute IC_50_s were obtained by probit analysis [22, 23] performed by fitting mean percentage inhibition values calculated from the growth data to the probit model by the maximum likelihood method [25] using SPSS Statistics 19 (IBM).

#### 3.3.3. 5-LO Products Biosynthesis Assays in HEK293 Cells

Cotransfection of HEK293 cells was performed using a pcDNA 3.1 vector expressing 5-LO and a pBUDCE4.1 vector expressing FLAP in presence of the Polyfect reagent (QIAGEN, Mississauga, ON, Canada) according to the manufacturer's protocol. Cell culture in presence of Geneticin and Zeocin (Invitrogen, Burlington, ON, Canada) generated stable double transfectants HEK 293 cells. The resulting stable double transfectants were propagated in culture and aliquots were frozen. Once thawed for a series of experiments, each aliquot of cells is cultured for a maximum of 6 weeks before being discarded. To stimulate the synthesis of 5-LO products, the transfected HEK 293 cells were collected and then trypsinized and washed and Hank's balanced salt solution (HBSS) containing 1.6 mM CaCl_2_ was used to resuspend the cell pellet at a concentration of 5 × 10^5^ cells mL^−1^. Each compound was preincubated at the indicated concentration with the cells for 5 min at 37°C and was followed by cell stimulation for 15 minutes at 37°C with the addition of 10 *μ*M calcium ionophore A23187 (Sigma-Aldrich, Oakville, ON, Canada) and 10 *μ*M arachidonic acid (Cayman Chemical, Ann Arbor, MI). Then, as described in a previous publication [12], stimulations were stopped and processed by RP-HPLC. Data are the means ± SEM of 3 independent experiments, each performed in duplicate.

#### 3.3.4. Assays of 5-LO Products Biosynthesis in Human PMNL Cells

Human PMNL were prepared from peripheral blood as described elsewhere [12] and were suspended in HBSS at 1.6 mM CaCl_2_ (10^7^ cells/mL) and prior to stimulation they were incubated with the test compounds for 5 min at 37°C in presence of 1 *μ*M thapsigargin [12]. Addition of 0.5 volume of cold MeOH : CH_3_CN (1 : 1) was used to stop the reactions and 50 ng of PGB_2_ was added as an internal standard. Samples were stored at −20°C until being analyzed by RP-HPLC (C_18_) column as indicated above. Data are means ± SEM of 3 independent experiments, each performed in duplicate (Figure 3).

### 3.4. Evidence of the Release of CAPE (**3**) by Salts **4** and **5**

#### 3.4.1. UV and HPLC Analysis

**Figure 4:**
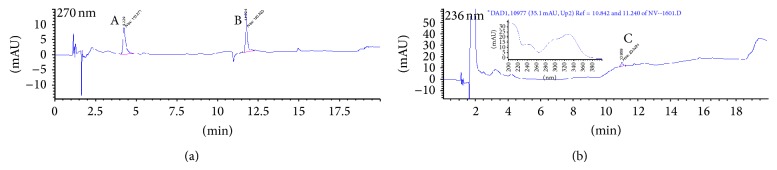
Chromatograms obtained for CAPE (**3**) at 270 nm (a) and 236 nm (b). Peaks are identified as follows: (A) 19-OH PGB_2_ (internal standard), (B) PGB_2_ (internal standard), and (C) CAPE (**3**). The inset in (b) shows the UV spectrum of peak C.

It is possible to track an inhibitor used in our 5-LO products biosynthesis bioassays if the inhibitor absorbs at the wavelengths set in the detection parameters of the diode array detector of the HPLC. The HPLC method used for tests with HEK293 and PMNL cells in the presence of our compounds was set for detection at 270 nm and 236 nm (as well as scanning peaks from 200 to 400 nm). To determine the release of CAPE (**3**) by sodium salt** 4** and ammonium salt** 5**, CAPE (**3**) was injected onto the HPLC system with the usual internal standards, without incubation in the presence of cells. The chromatograms obtained are shown in Figure 4. The UV spectrum associated with peak C (CAPE (**3**)) is presented in the inset of Figure 4.

**Figure 5:**
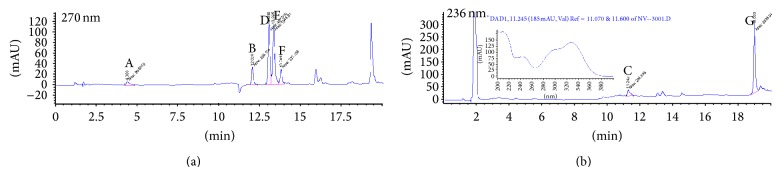
Typical chromatograms with detection at 270 nm (a) and 236 nm (b) obtained when stimulating HEK293 in the presence of 1 *μ*M of sodium salt** 4**. Peaks are identified as follows: (A) 19-OH PGB_2_ (internal standard), (B) PGB_2_ (internal standard), (C) CAPE (**3**) from sodium salt** 4**, as well as the 5-LO products, (D) 6-*trans* LTB_4_, (E) 6-*trans*-12-epi LTB_4_, (F) LTB_4_, and (G) 5-HETE. The inset in (b) shows the UV spectrum of peak C, which matches the CAPE (**3**) spectrum in Figure 4. All 5-LO products are checked against known standards (for retention time and UV spectral match).

**Figure 6:**
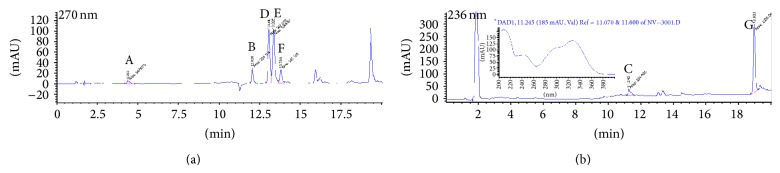
Typical chromatograms with detection at 270 nm (a) and 236 nm (b) obtained when stimulating HEK293 in the presence of 1 *μ*M of ammonium salt** 5**. Peaks are identified as follows: (A) 19-OH PGB_2_ (internal standard), (B) PGB_2_ (internal standard), (C) CAPE (**3**) from ammonium salt** 5**, as well as the 5-LO products, (D) 6-*trans* LTB_4_, (E) 6-*trans*-12-epi LTB_4_, (F) LTB_4_, and (G) 5-HETE. The inset in (b) shows the UV spectrum of peak C, which matches the CAPE (**3**) spectrum in Figure 4. All 5-LO products are checked against known standards (for retention time and UV spectral match).

Chromatographic analysis of HEK293 cells incubated with the sodium salt** 4** and ammonium salt** 5** shows the same peak as in Figure 4 (peak C). Figures 5 and 6 show the HPLC chromatograms obtained for extracts of HEK293 cells incubated with sodium salt** 4** and ammonium salt** 5**, respectively. The peak shows an absorption spectrum that is identical to that of CAPE. Peak C was absent in HEK293 cells incubated with the vehicle (DMSO) only. Peak C is also observed when CAPE (**3**) is used as the inhibitor in HEK293 cells and the same results are obtained when using PMNL cells (data not shown).

#### 3.4.2. NMR Analysis

To demonstrate the release of CAPE (**3**) by our salts, stability tests of** 5** in the presence of DMSO-d_6_ using NMR were performed. ^1^H and ^11^B NMR spectra are included in the Supplementary Material (see Supplementary Material available online at http://dx.doi.org/10.1155/2015/418362).

Compound** 5** (50 mg, 0.06 mmol) was dissolved in 1 mL of DMSO-d_6_ and the solution was analysed by multinuclear NMR spectroscopy after a period of 24 h. Spectroscopic NMR data: ^1^H (DMSO-d_6_); *δ* (ppm) 7.42 (d,* J* = 16.0 Hz, CHC_Ar_,** 5**), 7.36 (d,* J* = 15.8 Hz, CHC_Ar_,** 3**), 7.31–7.14 (ov m, Ar,** 3** and** 5**), 6.98 (d,* J* = 1.4 Hz, Ar,** 3**), 6.89–6.85 (ov m, H_Ar_,** 3** and** 5**), 6.82 (dd,* J* = 7.8, 1.4 Hz, Ar,** 5**), 6.70 (d,* J* = 8.1 Hz, H_Ar_,** 3**), 6.51 (d,* J* = 7.8 Hz, Ar,** 5**), 6.16 (d,* J* = 16.0 Hz, CHCO,** 3**), 6.15 (d,* J* = 15.8 Hz, CHCO,** 5**), 4.24 (t,* J* = 6.8 Hz, CH_2_O,** 5**), 4.10 (t,* J* = 6.8 Hz, CH_2_O,** 3**), 3.03 (br m, NCH_2_,** 5**), 2.91 (t,* J* = 6.8 Hz, OCH_2_CH_2_,** 5**), 2.90 (t,* J* = 6.8 Hz, OCH_2_CH_2_,** 3**), 1.45 (br m, –CH_2_–,** 5**), 1.20 (sext,* J* = 6.8 Hz, CH_2_CH_3_,** 5**), 0.82 (t,* J* = 6.8 Hz, CH_3_,** 5**). ^11^B (DMSO-d_6_): *δ* (ppm) 17.2 (B(OH)_3_), 12.5 (**5**). Stability tests for** 5** were performed using a Varian Mercury 200 Plus FT NMR spectrometer (^1^H: 200 MHz).

## 4. Conclusions

In summary, we have prepared new arylspiroborate salts from caffeic acid phenethyl ester and examined their antioxidant, 5-LO inhibition, and antimicrobial properties. Our results indicate that the prepared salts are indeed efficient carriers of CAPE whereby the antioxidant effects and the effects of inhibition of 5-LO were both increased compared to CAPE. There is also evidence that the sodium salt is more active than its corresponding ammonium salt, and this difference is probably due to the low solubility of the ammonium salt. The preliminary nature of this work meant that only a small library of analogues was tested; however, the observed biological activities of these compounds, especially their promising anti-inflammatory and antibacterial abilities, warrant further in-depth investigations into derivatives with enhanced solubility in aqueous media.

## Supplementary Material

NMR (^1^H and ^11^B) spectra of salt 5 can be found with the online version of this article.

## Figures and Tables

**Table 1:** Radical scavenging assay.

Compounds	IC_50_ (*μ*M) [SEM]^*^
Zileuton	>100
CAPE (**3**)	16.51 [4]
**4**	9.57 [1.35]
**5**	15.31 [1.95]

^*^Two independent experiments, each performed in duplicate.

**Table 2:** Antimicrobial activity (IC_50_s and MICs in *µ*M [mg/mL]) of boric acid and **3**–**5 **against *Candida albicans*, *Staphylococcus aureus,* and methicillin resistant *S. aureus*.

Compounds	*C*. *albicans *		*S*. *aureus *		MRSA	
	IC_50_; SEM^1^	MIC^2^	IC_50_; SEM	MIC	IC_50_; SEM	MIC
B(OH)_3_	Inactive^3^	Inactive	Inactive	Inactive	Inactive	Inactive

CAPE (**3**)	132.43; 0.40	176	39.1; 1.10	86	12.3; 1.50	86
	[37.65; 0.11]	[50]	[11.12; 0.32]	[25]	[3.5; 0.40]	[25]

**4**	72.2; 0.70	125	19.93; 1.45	42	22.9; 1.50	84
	[43.22; 0.40]	[75]	[11.93; 0.87]	[25]	[13.71; 0.88]	[50]

**5**	146.10; 1.50	>245	Inactive	Inactive	11.35; 1.10	61
	[119.50; 1.20]	[>200]			[9.28; 0.88]	[50]

^1^IC_50_s estimated by probit analysis. ^2^MIC (minimum inhibitory concentration) is defined as the lowest concentration that showed over 90% inhibition. ^3^Less than 50% inhibition in initial screening (at 100 *μ*g/mL) is considered to be inactive.

**Table 3:** 5-LO inhibition in PMNL cells.

Compounds	IC_50_ (*μ*M) [SEM]^*^
Zileuton	3.20 [0.3]
CAPE (**3**)	1.11 [0.03]
**4**	0.60 [0.05]
**5**	0.84 [0.03]

^*^Two independent experiments, each performed in duplicate.
