# Supplementary material for: Synthesis and Biological Activity of Arylspiroborate Salts Derived from Caffeic Acid Phenethyl Ester
Source: Int J Med Chem. 2015 Mar 5;2015:418362. doi: 10.1155/2015/418362 (PMC4365380; doi:10.1155/2015/418362)

dmso-d6
24 hours

STANDARD 1H OBSERVE

Archive directory: /export/home/student/vnmrsys/data
Sample directory: NBu4BCAPE2 24 hours_03Feb2015
File: PROTON

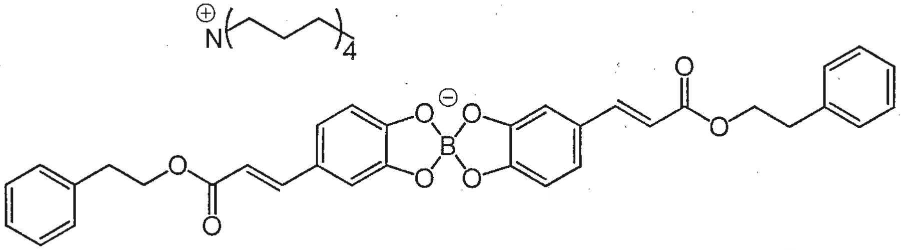

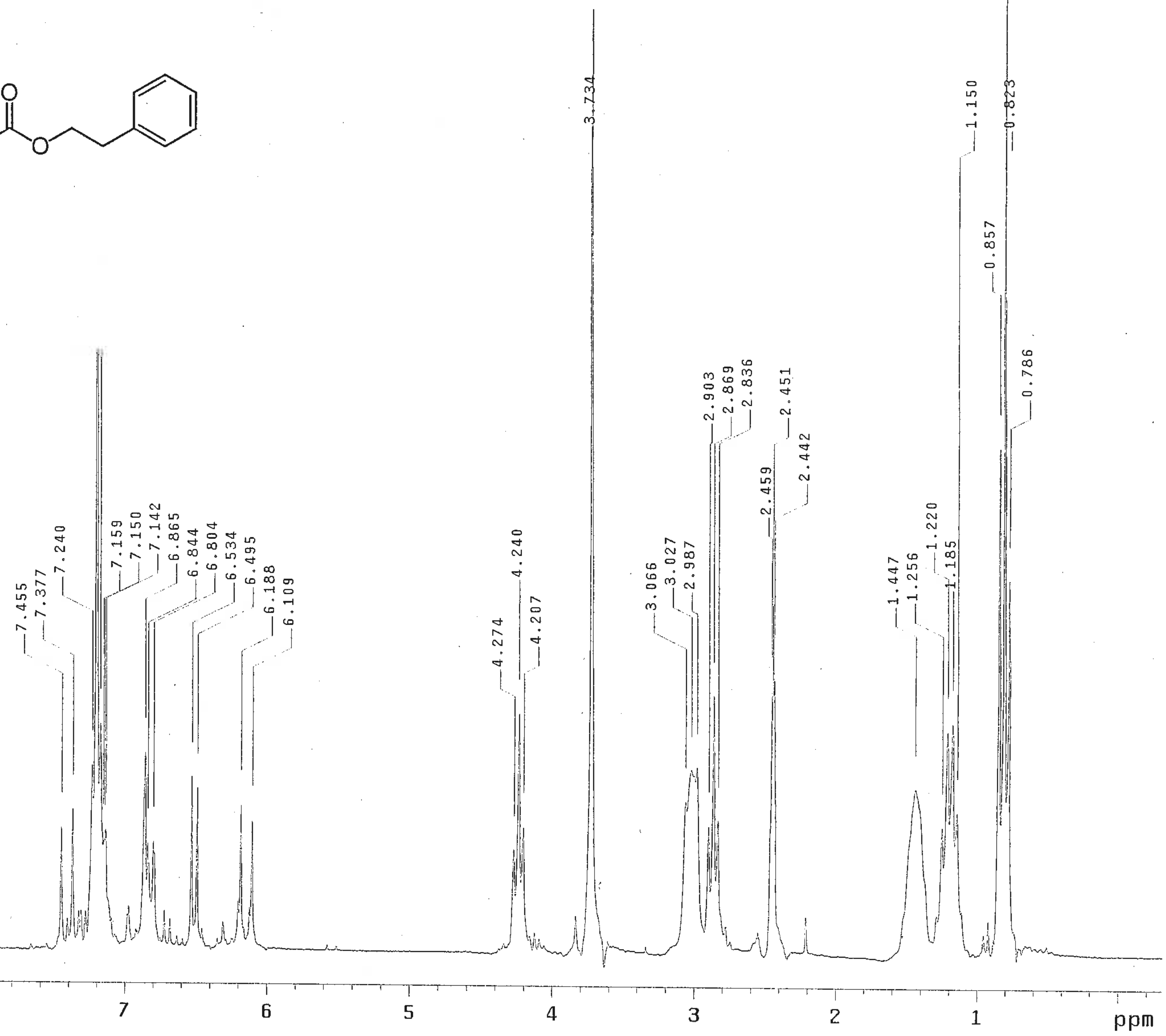

dmso-d6
24 hours

STANDARD 1H OBSERVE

Archive directory: /export/home/student/vnmrsys/data
Sample directory: NBu4BCAPE2 24 hours_03Feb2015
File: PROTON

Pulse Sequence: s2pul

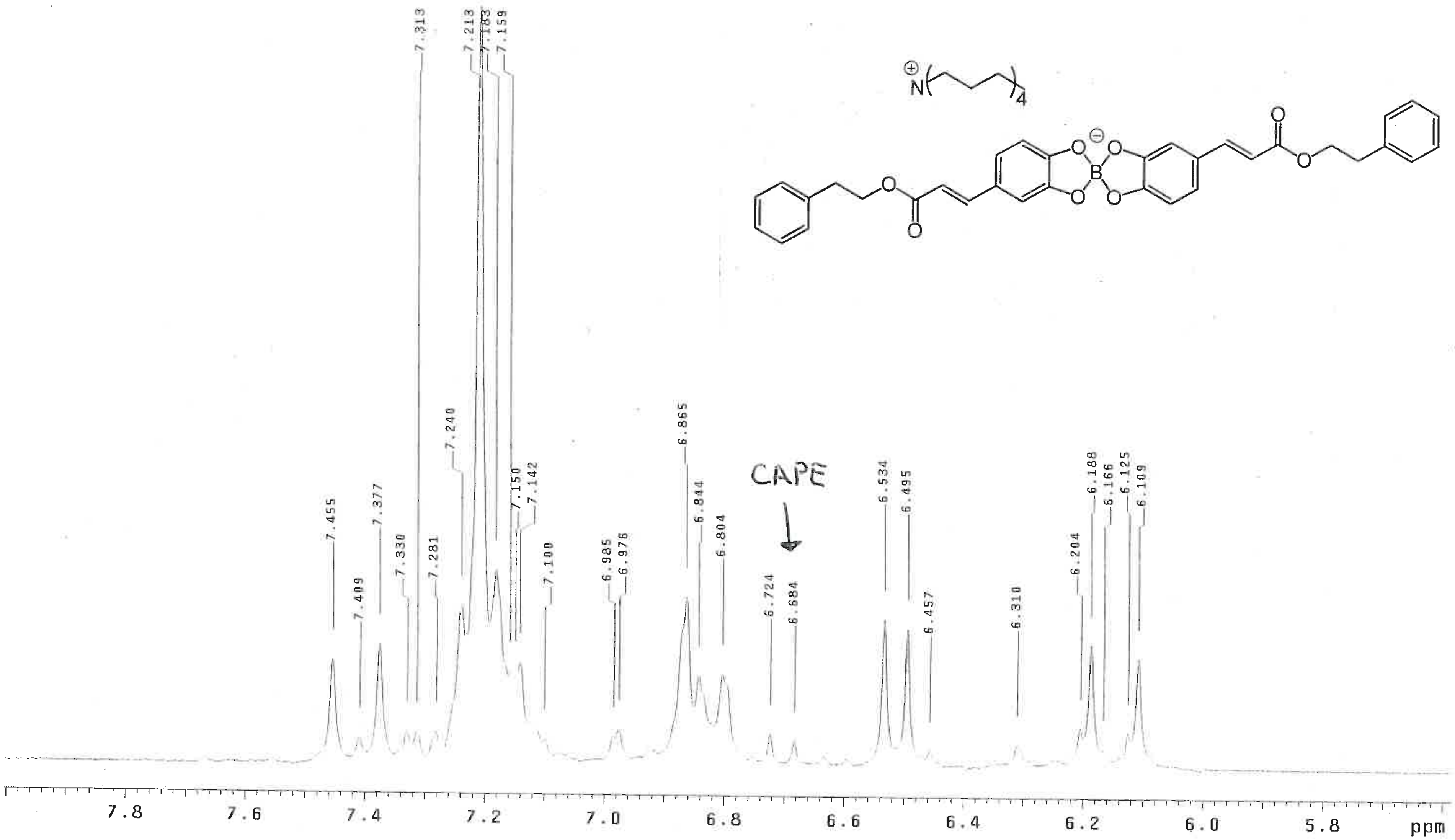

dmso-d_6
24 hours

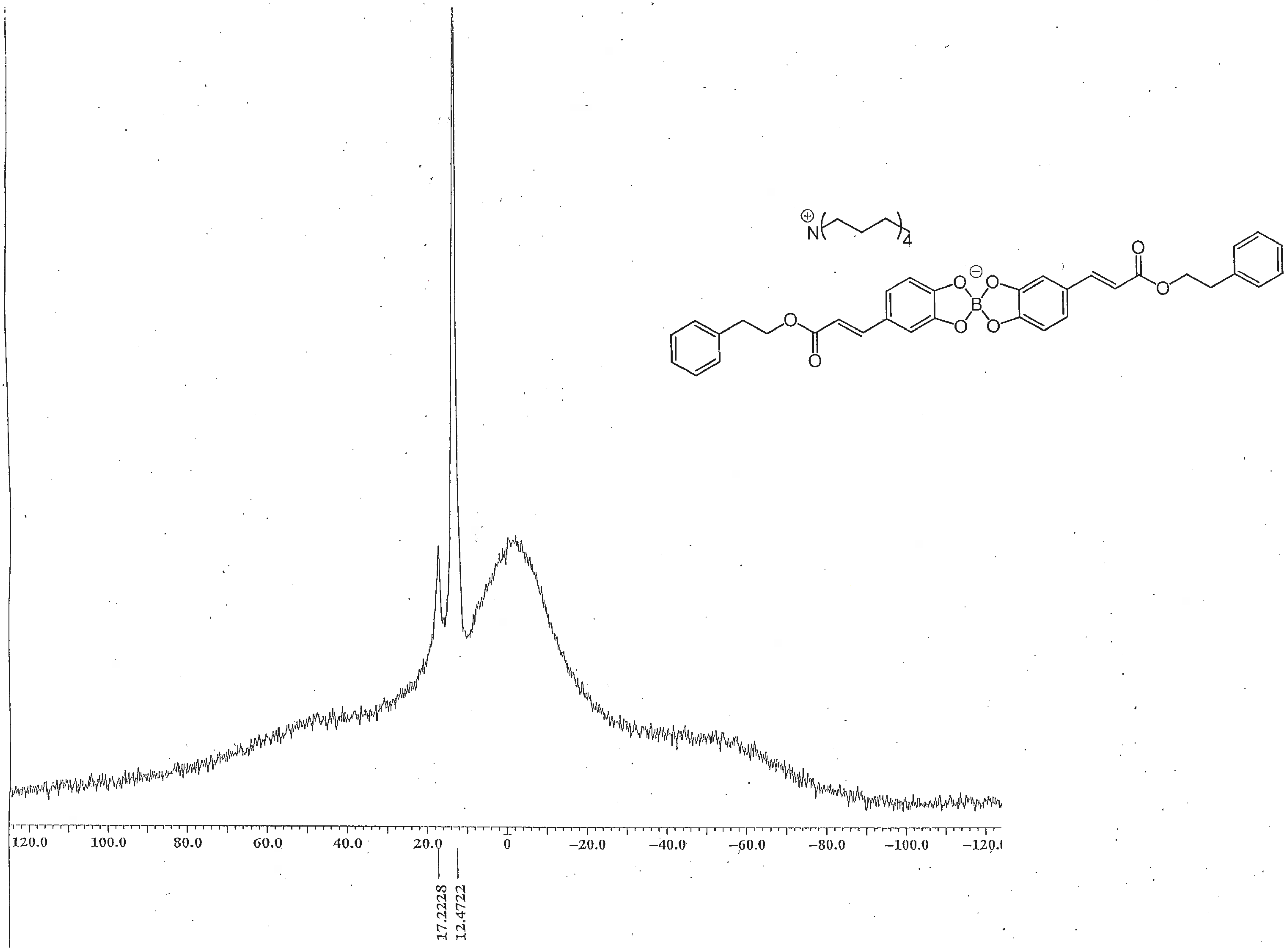

Supplement: Supplementary file 1 — NMR (1H and 11B) spectra of salt 5 can be found with the online version of this article. [file 418362.f1.pdf]
